# Clinical implementation of whole-genome array CGH as a first-tier test in 5080 pre and postnatal cases

**DOI:** 10.1186/1755-8166-4-12

**Published:** 2011-05-09

**Authors:** Sang-Jin Park, Eun Hye Jung, Ran-Suk Ryu, Hyun Woong Kang, Jung-Min Ko, Hyon J Kim, Chong Kun Cheon, Sang-Hyun Hwang, Ho-Young Kang

**Affiliations:** 1 MG MED, Inc., Seoul, Korea; 2 MACROGEN, Inc, Seoul, Korea; 3 Department of Medical Genetics, Ajou University School of Medicine, Suwon, Korea; 4 Department of Pediatrics, School of Medicine, Pusan National University Children’s Hospital, Yangsan, Korea; 5 Department of Laboratory Medicine, Center for Diagnostic Oncology, National Cancer Center, Gyeonggi-do, Korea

## Abstract

**Background:**

Array comparative genomic hybridization (CGH) is currently the most powerful method for detecting chromosomal alterations in pre and postnatal clinical cases. In this study, we developed a BAC based array CGH analysis platform for detecting whole genome DNA copy number changes including specific micro deletion and duplication chromosomal disorders. Additionally, we report our experience with the clinical implementation of our array CGH analysis platform. Array CGH was performed on 5080 pre and postnatal clinical samples from patients referred with a variety of clinical phenotypes.

**Results:**

A total of 4073 prenatal cases (4033 amniotic fluid and 40 chorionic villi specimens) and 1007 postnatal cases (407 peripheral blood and 600 cord blood) were studied with complete concordance between array CGH, karyotype and fluorescence *in situ *hybridization results. Among 75 positive prenatal cases with DNA copy number variations, 60 had an aneuploidy, seven had a deletion, and eight had a duplication. Among 39 positive postnatal cases samples, five had an aneuploidy, 23 had a deletion, and 11 had a duplication.

**Conclusions:**

This study demonstrates the utility of using our newly developed whole-genome array CGH as first-tier test in 5080 pre and postnatal cases. Array CGH has increased the ability to detect segmental deletion and duplication in patients with variable clinical features and is becoming a more powerful tool in pre and postnatal diagnostics.

## Background

Array comparative genomic hybridization (CGH) was developed as a genome wide screening strategy for detecting DNA copy number changes mainly in chromosomal disorders and cancer research [1-4]. Chromosomal abnormalities are a major cause of congenital and developmental abnormalities in human genetic diseases including dysmorphic features, mental retardation and developmental delay, and multiple congenital anomalies. Karyotype analysis has been the gold standard for pre and postnatal diagnosis. Using traditional cytogenetic techniques, chromosomal imbalances such as aneuploidies and segmental abnormalities must be larger than about 3~5 Mb to be detected by GTG banding. In the past two decades, various traditional banding techniques have been combined with molecular cytogenetic technologies to improve the resolution at which genomic changes can be detected. Array CGH technology has higher resolution and excellent throughput when compared to conventional and molecular cytogenetics. In array CGH, genomic DNA from the patient and reference are labeled with different fluorescent dyes and co-hybridized to an array matrix containing cloned DNA. The content of an array may include specific targeted regions of the genome or the entire genome arrayed on a single glass slide. Similar to conventional CGH, genome imbalances are quantified by analyzing the ratio of the two fluorescent hybridizing signals. The resolution of array CGH is determined by the size and number of the clones placed on the array to interrogate genome copy number changes. Whole genome or targeted array CGH is a powerful tool to accurately detect subtelomeric rearrangements. Array CGH has higher resolution and powerful clinical utility when compared with conventional and other molecular cytogenetic technologies. Array CGH has successfully detected DNA copy number changes, and several groups have studied the clinical application of this technology in both prenatal and postnatal samples [5-9]. Recently, The International Standard Cytogenomic Array (ISCA) Consortium reported consensus statement on the use of chromosomal microarray as a first-tier diagnostic test in the evaluation of individuals with developmental delays and/or congenital anomalies [10,11]. Our group successfully developed and validated a bacterial artificial chromosome (BAC) based array CGH analysis platform including analysis software [12]. This BAC array CGH analysis platform is based on 1440 fluorescence *in situ *hybridization (FISH) verified BAC clones that were selected among 100,000 BAC clones constructed by the Asian Genome Project [13] and validated by end-sequencing and FISH. This array CGH contains whole genomic regions including 356 major cell-growth related genes and over 40 known DNA copy number change disorders.

In this study, we applied our newly developed array CGH platform to 5080 clinical pre and postnatal cases and identified 114 abnormal cases with 75 prenatal and 39 postnatal cases. Our results support previous reports of the utility of array CGH for detecting chromosomal DNA copy number variations in prenatal and postnatal clinical cases.

## Materials and methods

### 1) Clinical samples

We analyzed the results obtained from 5080 clinical cases referred to MG MED laboratories for array CGH analysis between April 2007 and December 2009. We studied 4073 prenatal cases (4033 Amniotic Fluid [AF] and 40 Chorionic Villi [CV]) and 1007 postnatal cases (407 Peripheral Blood [PB] and 600 Cord Blood [CB]). The indications for the 4073 prenatal cases were family history, advanced maternal age, fetal ultrasound anomalies, elevated serum alpha fetoprotein, and parental anxiety. The indications for the 407 postnatal cases were mainly developmental delay, mental retardation, and multiple congenital abnormalities. The 600 postnatal cord blood samples were collected from the cord blood bank for detecting genomic imbalances, which were submitted by obstetricians, pediatricians, and geneticists. All samples were well prepared for experiments using previously described methods [12]. Appropriate ethical approval was obtained, and informed consent for the genetic testing was obtained from all patients.

### 2) Development of the array CGH analysis platform

We developed an BAC based array CGH analysis platform for detecting genomic imbalances in human genetic diseases [12]. Our array CGH chip consists of 1440 non-overlapping BAC clones (MACArray Karyo 1440 BAC-chip, Macrogen, Seoul, South Korea). A total of 1440 clone locations are shown on this website (http://www.macrogen.co.kr/eng/biochip/genelist_overview.html), which were selected among 100,000 BAC clones constructed by the Asian Genome Project [13,14] and carefully mapped, end-sequenced, and fluorescently labeled by FISH. All clones were two-end sequenced using an ABI PRISM 3700 DNA Analyzer (Applied Biosystems, Foster City, CA), and their sequences were Blast analyzed and mapped according to their positions as described in the University of California, Santa Cruz (UCSC) human genome database (http://www.genome.ucsc.edu). Confirmation of the locus specificity of the chosen clones was performed by removing multiple loci-binding clones by individual examination using standard FISH procedures as described previously.

### 3) Array CGH and data analysis

DNA was extracted from AF, CB, PB and CV using the PureGene kit (Gentra Systems, Minneapolis, MN). After extracting the DNA, we labeled 50~500 ng of both test and reference DNA with Cy 3- and Cy 5-dCTP (Perkin Elmer) by a random priming method using Exo-Klenow Fragment (Invitrogen, Carlsbad, CA) for 16 hr. labeled according to the manufacturer's recommendations. Reported here are pre and postnatal cases tested using the BAC chip. The array CGH chip data were analyzed using the chromofluor image analysis system (Array Analysis; Macrogen, South Korea). The slides contained 1440 human BAC clones including specific loci of more than 40 chromosomal disorders and 356 cell growth related genes from BAC libraries at a resolution average of 2.3 Mb for the entire genome. The human DNA source for making the BAC library was human sperm derived from a Korean man. Each BAC clone was represented on an array as triplicate spots, and each array was scanned using a GenePix4000B scanner (Axon Instruments, Foster City, CA, USA) and analyzed with array software (MAC VIEWER, Macrogen, South Korea). Green (test) to red (reference) (G/R) ratios were automatically determined for each sample, and the normalized G/R ratio represented the relative average number of copies of the sequence for those spots that were selected as controls. Spots with G/R ratios more than the mean plus 2.5 standard deviations (1.25) were considered amplifications or gains of the indicated copy number; less than the mean minus 2.5 deviations (-0.75) were considered losses of the copy number.

### 4) Karyotype and FISH with region-specific probes analyses

The chromosome analysis was performed according to standard methods using cultured cells from amniotic fluid and peripheral blood samples obtained from the patient and available parents. Metaphase preparations were analyzed by G-banding techniques. FISH studies on interphase or metaphase spreads with specific probes were performed following the manufacturer's protocols (Macrogen). FISH was performed with specific BAC clones to confirm the array CGH analysis. FISH was performed mainly with two color probes; the specific BAC probes labeled with Cy3 (red) and the control probe (green) with FITC. The specific BAC probes were constructed using BAC clone DNA (NCBI build35). FISH was performed on metaphase chromosomes cultured from amniotic fluid and peripheral blood lymphocytes from each patients and available parents. Image acquisition of metaphase cells and subsequent karyotyping were performed using the CytoVision system (Applied Imaging, Santa Clara, CA, USA). A karyotype was characterized according to the conventions of the International System for Human Cytogenetic Nomenclature (ISCN, 2009).

## Results

Array CGH is currently the most powerful method for simultaneously detecting genomic alterations. We performed whole genome array CGH using CGH array slides containing 1440 clones including about 40 chromosomal disorders specific loci and 356 cell growth related genes from BAC libraries in 5080 pre and postnatal cases (Table 1). We simultaneously performed FISH and a G-banding analysis to confirm abnormal array CGH results. In all prenatal cases, we performed array CGH analysis and concurrent karyotype analysis. We detailed genotype-phenotype correlations as far as possible in postnatal cases.

**Table 1:** Summary of array CGH analysis in 5080 cases

		Cases with abnormal array CGH analysis^a^			Total (N)	Detection rate (%)
				
		Aneuploidy (N)	Deletion (N)	Duplication (N)		
Prenatal cases^b ^(N = 4073)		60	7	8	75	1.8

Postnatal cases (N = 1007)	PB with clinical indications^c ^(N = 407)	5	19	10	34	8.3
	
	CB (N = 600)	0	4	1	5	0.83

Total	5080	65	30	19	114	2.24

PB, Peripheral Blood; CB, Cord Blood for banking in general population

^a^Karyotype and FISH analyses performed with array CGH anaysis. FISH analyses were performed using specific bacterial artificial chromosome (BAC) clones.

^b^AF, Amniotic Fluid (N = 4033); CV, Chorionic Villi (N = 40)

^c^Clinical indications; developmental delay, mental retardation, dysmorphic feature, multiple congenital anomalies, etc.

Of 4073 prenatal cases (4033 AF and 40 CV specimens), we identified 75 positive cases (75/4073 = 1.8%) with DNA copy number variations; 60 had an aneuploidy, seven had a deletion, and eight had a duplication (Table 2). Thirty-six cases had an autosomal aneuploidy, and 24 cases had a sex chromosome aneuploidy. The detection rate of overall chromosomal rearrangements including balanced translocations and inversions by karyotype analysis was 3.8% (155/4073). In prenatal cases, we identified deletion/micro deletion breakpoints (2q13, 7q11.23, 17p11.2, Xp22.31, and Xq24qter) and duplication/micro duplication chromosomes (1q42q44, 15q11.2q12, 21q11.2, Xp22.31, Xp21.2, and Xq27.2qter). A heterozygous micro deletion at 2q13, which includes the Joubert syndrome (2q13 homozygous deletion) critical region, was observed in two cases. The array CGH results were normal in two marker chromosome cases. The marker chromosomes seemed to consist of mainly heterochromatin or this may have been due to the limited array CGH coverage.

**Table 2:** Summary of array CGH and cytogenetic analyses in 4073 prenatal cases^a^

Case (number)	**Array CGH analysis**^b^	**Cytogenetic analyses**^c^
		Aneuploidy
1 (1)	Duplication of whole chr.13	Trisomy 13
2 (7)	Duplication of whole chr.18	Trisomy 18
3 (28)	Duplication of whole chr.21	Trisomy 21
4 (6)	Duplication of whole chr.X	47,XXX
5 (7)	Duplication of whole chr.X	47,XXY
6 (1)	Duplication of whole chr.X	Mos 47,XXY [18]/48,XXY,+17 [2]
7 (1)	Copy number ratio of less than one copy loss at chr. 9	Mos 47,XXX [29]/48,XXX,+9 [11]
8 (1)	Copy number ratio of less than one copy loss at chr.X	Mos 47,XXY [32]/46,XY [8]
9 (1)	Duplication of whole chr.X and 18	48,XXY,+18
10 (1)	Duplication of long arm at Xq10qter/whole chr. 9	47,X,i(X)(q10),+9
11 (4)	Deletion of whole chr.X	45,X
12 (1)	Copy number ratio of less than one copy loss at chr.X	Mos 45,X [22]/46,XX [8]
13 (1)	Copy number ratio of less than one copy loss at chr.X	Mos 45,X [10]/46,XX [20]
		Deletion/Microdeletion
14 (2)	Deletion of 0.5Mb at 2q13	46,XX,ish del(2)(q13q13)(NPHP1-)
15 (1)	Deletion of 0.4Mb at 7q11.23	46,XY.ish del(7)(q11.23q11.23)(ELN-)
16 (2)	Deletion of 0.4Mb at 17p11.2	46,XX.ish del(17)(p11.2p11.2)(D17S29-)
	Deletion of 0.4Mb at 17p11.2	46,XY.ish del(17)(p11.2p11.2)(D17S29-)
17 (1)	Deletion of 0.5Mb at Xp22.31	46,XY.ish del(X)(p22.31p22.31)(STS-)
18 (1)	Deletion of 25Mb at Xq24qter	46,X,del(X)(q24qter)
		Duplication/Microduplication
19 (1)	Duplication of 10Mb at 1q42q44	46,XY.ish dup(1)(q42.1q44)(D1S491+)
20 (2)	Duplication of 1.3Mb at 15q11.2q12	46,XX.ish dup(15)(q11.2q12)(SNRPN+)
21 (2)	Duplication of 1.5Mb at 22q11.2	46,XY.ish dup(22)(q11.2q11.2)(COMT+)
22 (1)	Duplication of 0.5Mb at Xp22.31	46,XX.ish dup(X)(p22.31p22.31)(KAL1+)
23 (1)	Duplication of 0.5Mb at Xp21.2	46,XY.ish dup(X)(p21.2p21.2)(DMD+)
24 (1)	Duplication of 5Mb at Xq27.2qter.	46,XY,ish dup(X)(q27.2qter)(DX904+)
		Small supernumerary marker chromosome
25 (2)	Normal	47,XX,+mar
	Normal	47,XY,+mar
26 (80)	Normal	Others^d^

^a^Data compiled from array CGH and cytogenetic analyses in 4033 AF and 40 CV by the major indications for prenatal testing such as advanced maternal age, fetal ultrasound anomalies, and elevated serum alpha fetoprotein .^b^log_2 _mean green/red (G/R) ratios more than the mean + 2.5 SD (~ 0.25) were considered high amplifications or gains of the indicated copy number, and less than the mean -2.5 SD (~ -2.5) were considered high losses of the copy number.^c^performed by karyotype and FISH analyses. FISH analyses were performed using specific BAC clones. ^d^Structural balanced arrangements (inversion and translocation).

**Figure 1:**
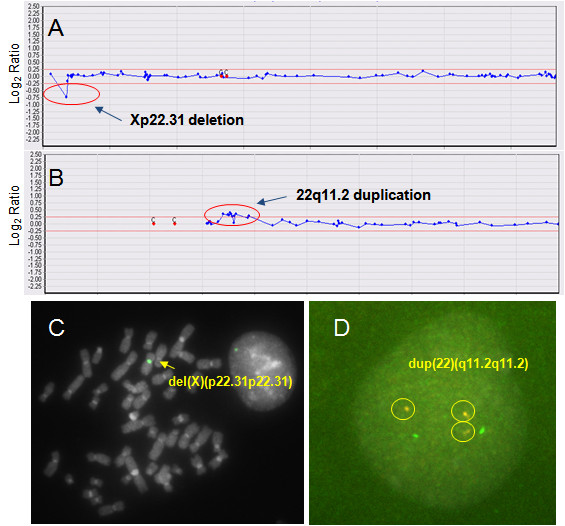
**Array CGH with FISH validation data for cases 17 (A, C) and 21 (B, D) (Table 2)**. (A) The array CGH results for the X chromosome. Arrow indicates deletion of the steroid sulfatase deficiency critical region (Xp22.31) including the *STS *gene. (B) The array CGH results for chromosome 22. Arrow indicates duplication of the Digeorge syndrome critical region (22q11.2). (C) FISH with a Xp22.31 specific region probe; arrow indicates a deletion of the probe (STS-) in a del(X)(p22.31p22.31) chromosome. (D) FISH with 22q11.2 specific region probe; circles indicate a duplication of the probe (COMTⅹ3) in an interphase cell.

**Figure 2:**
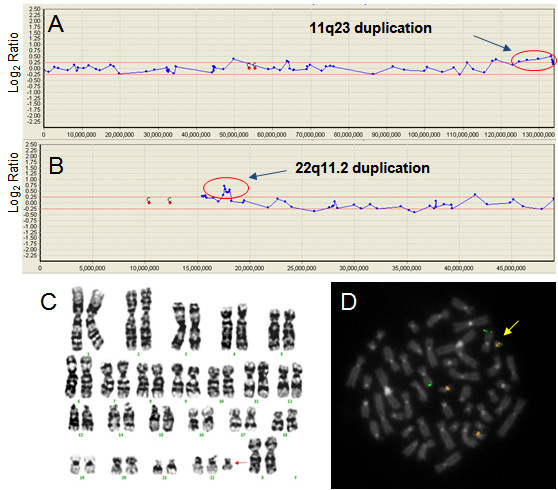
**Array CGH with cytogenetics validation data for case 30 (Table 3)**. (A), (B) The array CGH results for chromosomes 11 and 22. Arrows indicates duplication of 11q23 and 22q11.2. (C) G-banding karyotype result of 47,XX, +der(22)t(11;22)(q23;q11). Arrow indicates the marker chromosome. (D) FISH with 11q23 (green color) and 22q11.2 (yellow color) specific region probes; arrow indicates a +der(22)t(11;22)(q23;q11).

A total of 1007 postnatal cases (407 PB and 600 CB) were studied with complete concordance between array CGH, karyotype and FISH results. Among the postnatal cases with 39 positive cases, five had an aneuploidy, 23 had a deletion, and 11 had a duplication (Table 3). We identified breakpoints on 17 types of deletion/micro deletion and seven types of duplication/micro duplication chromosomes. The most common indications for referral in the 407 PB cases were developmental delay, mental retardation, congenital abnormalities, and dysmorphic features. We identified 34 deletion/duplication cases (34/407 = 8.3%) in 407 PB cases with clinical indications. In 600 CB cases for screening genomic imbalances from the cord blood bank, we found four deletion (15q11.2, 22q11.2, and Xp11.2petr) cases and one duplication (2q13) case (5/600 = 0.83%). Thirty-four cases had segmental gains or losses associated with chromosomal deletion (micro deletion) or duplication (micro duplication), including Wolf-Hirschhorn syndrome (WHS)(4p16.3), Cri-du-chat syndrome (5p15.3), Soto's syndrome (5q35.3), William's syndrome (7q11.23), Digeorge syndrome 2 (10p14), Prader Willi/Angelman syndrome (15q11.2), Digeorge syndrome (22q11.2), steroid sulfatase deficiency (Xp22.31), Cat eye syndrome (22q11.2), and Emanuel syndrome (22q11, 11q23). We performed array CGH, and the molecular cytogenetic results are shown in Figure 1 (examples of prenatal deletion/duplication cases) and 2 (examples of postnatal deletion/duplication cases). We also found clinical features in the postnatal cases. For example, the postnatal case 10 patient with Sotos syndrome was a 1.1-year-old male with a birth weight of 4.1 kg. He had typical facial features including macrocephaly, sparseness of frontotemporal hair, high bossed forehead, a long narrow face and prominent narrow jaw, developmental delay, overgrowth, abnormally large hands, and a café au lait spot. We identified not only simple deletion and duplication but also multiple copy number abnormalities including various chromosomal rearrangements (postnatal cases 4, 24, 27, and 30). Additionally, postnatal case 4 was identified with multiple rearrangements. Although we found a 2q14q21.1 deletion using array CGH, we were able to characterize accurate cytogenetic features as 46,XY, der(2)t(2;6)(q21.1;q23)inv(2)(q14.1q37) and der(6)t(2;6)(q21.1;q23) by concurrent karyotype analysis (data not shown). In case 30, we identified two chromosomes involved as a small supernumerary marker chromosome (sSMC) as 47,XX, +der(22)t(11;22)(q23;q11) with mental retardation (Figure 2).

**Table 3:** Summary of array CGH and cytogenetic analyses in 1007 postnatal cases^a^

Case (number)	**Array CGH analysis**^b^	**Cytogenetic analyses**^c^	Involved Gene(s)	Clinical indications
		Aneuploidy		
1 (3)	Duplication of whole chr.21	Trisomy 21		DD,MR
2	Duplication of whole chr.X	47,XXY		Klinefelter's syndrome
3	Duplication of whole chr.Y	47,XYY		
		Deletion/Microdeletion		
4	Deletion of 15Mb at 2q14q21.1	46,XY, der(2)t(2;6)(q21.1;q23)	Multiple	DD,MR
		inv(2)(q14.1q37), der(6)t(2;6)(q21.1;q23)		
5	Deletion of 1Mb at 3q23q25	46,XY. ish del(3)(q23q25)(D3S1557-)	ZIC1,4	DD,IA, CP
6	Deletion of 0.5Mb at 3q29	46,XY, ish del(3)(q29q29)(MF12-)	PAK2,DLG1	DD
7	Deletion of 1Mb at 4p16.3	46,XX, del(4)(p16.3p16.3)	WHSC1	WHS
8	Deletion of 0.8Mb at 4q35.1qter	46,XX.ish del(4)(q35.1qter)(D4S187-)	Multiple	DD
9(2)	Deletion of 0.8Mb at 5p15.3	46,XY.ish del(5)(p15.3p15.3)(D5S2774-)	Multiple	DD,MR
10	Deletion of 0.5Mb at 5q35.2	46,XY.ish del(5)(q35.2q35.2)(NSD1-)	NSD1	Sotos syndrome
11(2)	Deletion of 0.4Mb at 7q11.23	46,XY.ish del(7)(q11.23q11.23)(ELN-)	ELN	Williams syndrome
12	Deletion of 5Mb at 10p12.4p14	46,XY. ish del(10)(p12.4p14)(D10S585-)	NEBL	DGS2
13	Deletion of 0.5Mb at 12q14.3	46,XY.ish del(12)(q14.3q14.3)(D12S1448- )	LEMD3	DD
14	Deletion of 5Mb at 14q32.2qter	46,XX.ish del(14)(q32.2qter)(SHGC172944-)	Multiple	DD
15(2)	Deletion of 0.5Mb at 15q11.2q11.2	46,XY.ish del(15)(q11.2q11.2)(SNRPN-)	SNRPN	PWS
16	Deletion of 0.4Mb at 17p11.2	46,XY.ish del(17)(p11.2p11.2)(PMP22-)	PMP22	
17	Deletion of 0.8Mb at 18p11.32	46,XX, ring(18)(p11.32q23)	Multiple	DD
18 (2)	Deletion of 2.5Mb at 22q11.2	46,XY.ish del(22)(q11.2q11.2)(D22S75-)	TBX1	DGS
19 (3)	Deletion of 0.5Mb at Xp22.31	46,X.ish del(X)(p22.31p22.31)(STS-)	STS	ichthyosis, ADHD
20	Deletion of 1Mb at Yq11.2qter	46,X.ish del(Y)(q11.2qter)(CDY1-)	CDY1	Azoospermia
		Duplication/Microduplication		
21	Duplication of 5Mb at 1q42.2qter	46,XY.ish dup(1)(q42.2qter)(D1S204+)	Multiple	
22	Duplication of 0.5Mb at 2q13	46,XX.ish dup(2)(q13q13)(NPHP1+)	NPHP1	
23	Duplication of 3Mb at 15q11.2q12	46,XX.ish dup(15)(q11.2q12)(SNRPN+)	SNRPN	DD,Autism,PD
24	Duplication of 0.9Mb at 21q22 & Deletion of 0.5Mb at 21q22	46,XY.ish del(21)(q22q22), dup(21)(q22q22)(D21S1898+)	Multiple	DD
25 (2)	Duplication of 0.9Mb at 22q11.2	46,XY.ish dup(22)(q11.2q11.2)(D22S75+)	TBX1	DD
26	Duplication of 0.9Mb at Yp11.2pter	46,X.ish i(Y)(p11.2pter)(DYS289+)	SRY	Azoospermia
27	Duplication of 3Mb at Yq11.2qter &	46,X.ish del(X)(p22.31p22.31),	STS	Short stature,ADHD
	Deletion of 0.5Mb at Xp22.31	dup(Y)(q11.2qter)(STS-,CDY1+)	CDY1	
		Small supernumerary marker chromosome		
28	Duplication of 3Mb at 18p11.2p11.3	47,XY,+der(18)(p11.2p11.32)	Multiple	DD
29	Duplication of 0.9Mb at 22q11.2qter	47,XY,+mar.ish i(22)(q11.2qter)(D22S43+)	Multiple	Cat eye syndrome
30	Duplication of 2Mb at 22q11& 3Mb at 11q23	47,XX,+mar.ish +der(22)t(11;22)(q23;q11) (CES+,D11S4145+)	Multiple	Emanuel syndrome

DD, developmental delay; MR, mental retardation; IA, imperforated anus; CP, cleft palate; WHS, Wolf-Hirschhorn syndrome; DGS, DiGeorge syndrome; PWS, Prader-Willi syndrome; ADHD, Attention Deficit Hyperactivity Disorder; PD, Pigmentation disorder

^a^Data compiled from 407 PB and 600 CB.

^b^log_2 _mean green/red ratios more than the mean +2.5 SD (~ 0.25) were considered high amplifications or gains of the indicated copy number, and less than the mean -2.5 SD (~ -0.25) were considered high losses of the copy number.

^c^performed by karyotype and FISH analyses. FISH analyses were performed using specific BAC clones.

## Discussion

Array CGH offers a high-resolution genome analysis in a clinical setting [15]. Currently, many commercial and academic laboratories have used BAC-based array, oligonucleotide-based array or SNP-based arrays. Array resolution is dependent on the number and types of probes used and how they are designed on the genome. Current commercial BAC arrays (targeted or whole genome) generally have 600~5200 BAC clones (e.g. PerkinElmer). Our whole-genome BAC array CGH platform is based on 1440 fluorescence *in situ *hybridization (FISH) verified BAC clones, which were selected among 100,000 BAC clones constructed by the Asian Genome Project [13,14] and validated by end-sequencing and FISH. Therefore, our abnormal array CGH results were able to be confirmed by FISH, that BAC clones were available to clinically relevant turnaround times.

In this study, we showed the utility of our whole-genome BAC-based array CGH platform in a large collection of clinical cases. Previous prenatal studies reported chromosomal abnormalities in ~1.3% of cases [16]. Furthermore, previous postnatal studies have shown that array CGH with cytogenetic analysis detects clinically significant chromosome abnormalities in ~7% of children referred for cytogenetic testing [16]. In this study, the prenatal detection rate was 1.8% (75/4073) and the postnatal detection rate with clinical indications was 8.3% (34/407) using array CGH (Table 1). These results are consistent with previous reports [17]. In prenatal cases, we identified 11 types with DNA copy number changes including five micro deletions and six micro duplications. Because these micro deletion/duplication chromosomal abnormalities could not be detected by conventional cytogenetic methods, array CGH analysis was more useful. We also classified the pathogenic variants. In case 14 (deletion at 2q13), we identified a heterozygous deletion using FISH analysis. Homozygous deletions of this locus (2q13 deletion) are pathogenic variants related to Joubert syndrome [18]. We also identified 15q11.2 and 22q11.2 duplications (cases 20 and 21). These duplication copy number variation phenotypes appear to be generally mild and highly variable; findings ranged from apparently normal to mental retardation, growth retardation, and autism (15q11.2 duplication). Therefore, prenatal array CGH results must be evaluated whether the results show benign variants or pathogenic variants. Currently, many useful copy number variation data base websites are available. For example, the Database of Genomic Variants (http://projects.tcag.ca/variation/), DECIPHER (http://decipher.sanger.ac.uk/), and Genereviews (http://www.genetests.org/), which is a more clinically useful website that lists the associated genetic diseases including phenotypes, genotypes, and clinical management of pathogenic copy number variant diseases. Many known micro deletion syndromes are expressed by incomplete penetrance [19], whereas duplications generally produce milder and, therefore, less identifiable phenotypes than counterpart deletions [20]. A previous study suggested an effective general algorithm for clinical testing using array CGH [21]. Moreover, practical guidelines for array CGH have been issued by the American College of Medical Genetics [22,23]. It is important that, with the exception of purported benign copy number variables (CNVs), all regions showing abnormal copy number findings be characterized by FISH and parental studies. An increase in the prenatal detection rate of chromosome abnormalities would benefit patients seeking genetic testing prior to delivery [24,25].

**Figure 3:**
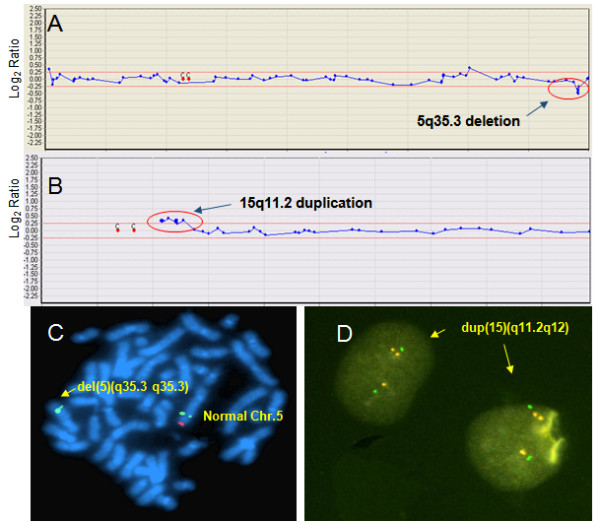
**Array CGH with FISH validation data for cases 10 (A, C) and 23 (B, D) (Table 3)**. (A) The array CGH results for chromosome 5. Arrow indicates deletion of the Sotos syndrome critical region (5q35.3), including the *NSD1 *gene. (B) The array CGH results for chromosome 15. Arrow indicates duplication of the PWS/AS syndrome critical region (15q11.2). (C) FISH with a 5q35.3 specific region probe; arrow indicates a deletion of the probe (NSD1-) in a del(5)(q35.3q35.3) chromosome. Deletion of the NSD1 gene region (red signal) was observed by FISH analysis, 46, XY, ish del(5)(q35.3q35.3)(D5S404+, NSD1-) (D) FISH with 15q11.2 specific region probe; arrows indicate a duplication of the probe (SNRPNⅹ3) in interphase cells.

In postnatal cases, we identified breakpoints on 17 types of deletion/micro deletion, seven types of duplication/micro duplication chromosomes and three sSMC types. Among the 39 positive postnatal cases, five had an aneuploidy, 23 had a deletion, and 11 had a duplication (Table 3). Most postnatal cases had clinical indications with developmental delay or mental retardation. Therefore, detection rates of chromosomal abnormalities using array CGH analysis are generally higher than prenatal cases. In this study, we analyzed various patients with clinical indications and/or unknown specific indications. We observed a 3q24 deletion in a patient (case 5) with developmental delay, imperforated anus, and cleft palate. Deletion of the 3q24-q26 region including the Dandy-Walker malformations (DWM) critical region (3q24) appears to be associated with a somewhat similar constellation of findings including craniofacial dysmorphism (broad and depressed nasal bridge and low set posteriorly rotated ears), mental retardation, congenital heart defects, and central nervous system malformations [26]. A previous study reported the critical region associated with DWM, which encompasses the ZIC1 and ZIC4 genes, by mapping the 3q24 interstitial deletions in several individuals with DWM [27]. In case 10, we identified a micro deletion of the 5q35 region including the *NSD1 *gene in one Korean patient with Sotos syndrome (Figure 3). The most common mutation (50%) was a micro deletion of the 5q35 region including *NSD1 *in Japanese patients with Sotos syndrome, while, in the UK, approximately 70% of patients with Soto syndrome have *NSD1 *point mutations, and only 10% of the patients have a micro deletion of the 5q35 region [28]. Therefore, further studies will be necessary for a better understanding of Sotos syndrome in Korean patients. The most commonly observed deletions were in the Xp22.31 region. Our patients with Xp22.31 (cases 19 and 27) appeared to be associated with ichthyosis, short stature, and attention deficit hyperactivity disorder (ADHD). The Xp22.31 deletion, causing loss of function of the STS gene, is associated with steroid sulfatase deficiency in males. The STS gene encodes steroid sulfatase, a membrane-bound microsomal enzyme that is ubiquitously expressed and hydrolyzes several 3-beta-hydroxysteroid sulfates, which serve as metabolic precursors for estrogens, androgens, and cholesterol [29]. A previous study reported on a patient with a Xp22.3 interstitial deletion that had ichthyosis, dysmorphic features, and mental retardation with ADHD [30]. We identified duplication cases at 15q11.2q12 with developmental delay, autism, and pigmentation disorder (Figure 3). A previous study reported that this patient had infantile autism with cytogenetic abnormalities on chromosomal region 15q11-q13, as reported in patients with autistic disorder [31]. We identified three marker chromosome origins (11, 18, and 22 chromosome segments). sSMC origin analysis is very important for evaluating clinical phenotypes. The marker chromosome origin and size appear to result in variable phenotypes [32]. In our study, we identified a 22q11.2 duplication including the CES gene and two chromosomes involved in derivative translocation marker chromosome (22q11&11q23 duplication) (Figure 2). This sSMC, der(22)t(11;22)(q23;q11), is related to Emanuel syndrome, which is characterized by severe mental retardation, microcephaly, failure to thrive, preauricular tag or sinus, ear anomalies, and cleft or high-arched palate [33]. These array CGH and other cytogenetic findings are consistent with the clinical phenotypes of Cat eye syndrome and Emmanuel syndrome with mental retardation. We also identified CNVs (2q13 duplication and 4q32 duplication) in phenotypically normal individuals from cord bank blood. The clinical significance of the 2q13 duplication is still emerging, as these copy number variations are also found in phenotypically normal and control individuals [34]. Duplication of 4q32 is a benign copy number variation represented in a copy number variation database (http://projects.tcag.ca/variation/).

And also, it is important to address some of the limitations of array CGH before this test is considered for clinical diagnosis as a first line test. Array CGH does not detect polyploidy, balanced translocations, inversions and low level mosaicims. Marker chromosomes may also be missed, depending on the size, marker composition and array coverage of the specific chromosomal region present on the marker chromosome. And also we must consider various limitations of array CGH testing (e.g. point mutations, uniparental disomy and appropriate turnaround times). Therefore, genetics laboratories must be capable of performing array CGH and concurrent conventional cytogenetic analyses (G-banding and FISH) including molecular genetic analyses (DNA sequencing etc.) and also clinical geneticists offer appropriate genetic counseling including interpretation of results and limitations of test for patients and family members.

## Conclusions

This study demonstrated that our newly developed array CGH platform is very useful for clinical implementation using whole-genome array CGH as first-tier test in 5080 pre and postnatal cases. The application of array CGH with more extended coverage at disease specific regions and concurrent appropriate other cytogenetic analyses such as karyotype and FISH will be useful to characterize various chromosomal disorders. Furthermore, the newly developed array CGH analysis platform will lead to a new understanding of genomic disorders with DNA copy number changes and their relationship to genotype and phenotype. Additionally, functional studies based on the identity of the involved genes are necessary for a further understanding of the mechanism related to contiguous genes on deletion and duplication loci in the development and/or progression of various DNA copy number change disorders.

## Competing interest statement

The authors declare that they have no competing interests.

## Authors' contributions

SJP drafted the manuscript and analysed the data for the paper. EHJ performed array CGH. RSR performed karyotyping and FISH. HWK performed FISH, relevant confirmations and participated in validation work on the BAC array CGH platform. JMK, HJK, CKC and SHH provided clinical information, evaluation and advice. HYK conceived of the study, performed data analysis and also approved the manuscript. All authors read and approved the final manuscript.
